# Signal-to-event encoding parameter selection for multiple event classification with spiking neural networks

**DOI:** 10.3389/fnins.2025.1610766

**Published:** 2025-06-23

**Authors:** Mateusz Pabian, Dominik Rzepka, Mirosław Pawlak, Marek Miśkowicz, Ryszard Sroka

**Affiliations:** ^1^Department of Measurement and Electronics, AGH University of Krakow, Kraków, Poland; ^2^Department of Electrical and Computer Engineering, University of Manitoba, Winnipeg, MB, Canada

**Keywords:** event-based signal encoding, van Rossum distance, Bayesian optimization, multiple event classification, spiking neural networks, k-NN classifier

## Abstract

Event-driven systems can operate either on discrete-time event streams or on analog signals transformed into the event domain by a predefined encoding scheme. This paper studies the problem of optimal event-based signal encoding if data are to be processed by a machine learning model, such as the spiking neural network (SNN). We introduce a method of encoding parameter selection that evaluates a k-Nearest Neighbor (k-NN) classifier operating on a measure of the event stream distance in multiple trials of a Bayesian optimization process. The efficiency of the proposed method is assessed by relating the classification performance with the number of events produced by a signal-to-event encoding scheme. The proposed method is validated for vehicle monitoring sensor data with three event-based encoding schemes: level-crossing encoding, send-on-delta, and leaky integrate-and-fire encoder. The best-performing sets of encoding parameters give an average accuracy of up to 0.912 for the k-NN classification, while producing 97.8% fewer number of samples than for the classical periodic discrete-time signal representation. Additionally, we train the SNN classifiers on data encoded according to the selected sets of parameters, achieving an average classification accuracy of up to 0.946, improving upon the k-NN baseline. This shows that the proposed model-agnostic signal-to-event encoding parameter selection is promising for training sophisticated machine learning models.

## 1 Introduction

Event sequence data is a natural representation for modeling of discrete-event systems, in which the state changes occur at discrete points in time due to specific events (Cassandras and Lafortune, 2008). The discrete-event systems (DESs) are commonly used to model and analyze processes that evolve in steps such as the arrival of a data packet, completion of a task, or a request for service. The examples of DESs are manufacturing and computer operating systems (banking, social media, etc.), as well as telecommunication or healthcare systems.

The event representation can also be applied to continuous dynamics present in event-based control and signal processing (Heemels et al., 2012; Tsividis, 2003; Miśkowicz, 2015), especially if implemented within the Industrial Internet of Things (IIoT) (Aranda-Escolástico et al., 2024). Event-based representations of continuous-time processes focus on capturing key moments in the system evolution, when significant changes—considered “events”—occur. The event rate for a given continuous input is usually much lower than when that input is represented at regular time intervals (Miśkowicz, 2003). This helps to reduce data transmission and energy consumption in applications such as environmental monitoring, industrial process automation, smart grids and power systems (Aranda-Escolástico et al., 2024). Event-based techniques enable resource-aware design and are especially effective in resource-constrained applications.

The classical event-based representations of continuous-time signals are level-crossing models where events are produced when a signal crosses a specific threshold or a set of levels (Mark and Todd, 1981), or send-on-delta reporting when events are triggered by signal changes by a prespecified increment (Miśkowicz, 2006). The send-on-delta encoding is used in event-triggered control (Miśkowicz, 2015) and state estimation (Ge et al., 2020), as well as in biologically-inspired event sensors that mimic the function of eyes, ears, nose, or touch (Tayarani-Najaran and Schmuker, 2021; Cheng et al., 2019). On the other hand, the level-crossing representations are applied in event-based signal processing (Miśkowicz, 2015) including signal reconstruction (Rzepka et al., 2018), bandwidth estimation (Rzepka et al., 2017), ECG signal analysis (Ravanshad et al., 2013), design of analog-to-digital converters (Ravanshad et al., 2013; Wang et al., 2018), and digital filters (Huber and Liu, 2019).

Some event-based representations are biologically inspired. One example is the integrate-and-fire encoder (Stein, 1965; Thao et al., 2023), that simplifies real neuron dynamics by mimicking two essential behaviors: integration as a sum of incoming electrical inputs over time, and firing as a generation of spike (event) when the input reaches a certain threshold. The integrate-and-fire model is frequently used in computational neuroscience and machine learning to simulate the behavior of biological neurons in spiking neural networks (SNNs) (Nunes et al., 2022). This enables networks to process information based on the timing of events (spikes) rather than continuous values, offering advantages for temporal data analysis, especially for time-series data or sensory processing tasks.

This paper explores the problem of optimal event-based signal encoding for further processing of event streams by classification models. The proposed approach involves fitting a k-NN classifier to an event stream using the van Rossum distance measure through multiple trials in a Bayesian optimization process. Based on the results of Bayesian optimization, we determine a fixed set of signal-to-event encoding parameters suitable for further processing by more advanced classification techniques.

To validate the proposed methodology, we use sensor data from an Intelligent Transportation System (ITS) for vehicle monitoring (Marszałek et al., 2023). Our method makes no assumptions about the underlying signal, ensuring its adaptability to other data domains. To demonstrate how the encoding parameters selected by the k-NN classifier can support multiple event classifications, we train a time-to-first-spike spiking neural network (SNN) (Mostafa, 2018) on event-data streams. The efficiency of the proposed method is evaluated by analyzing the relationship between classification performance and the number of events generated by the event-based encoding scheme. This allows us to assess the impact of signal- to-event encoding on classification accuracy for this dataset.

This paper is structured as follows. Section 2 provides a brief overview of event-based signal encoding schemes, spiking neural network training methods, and introduces the technical context of the vehicle classification problem used to evaluate the proposed methodology. Section 3.1 presents the paper's main contribution: a model-agnostic method for selecting signal-to-event encoding parameters. Section 3.2 focuses on the time-to-first-spike SNN model trained on event data encoded according to the proposed signal-to-event encoding schemes. Section 4 details an extensive simulation study assessing the methodology in the context of the vehicle classification problem. Finally, Section 5 summarizes the findings and provides recommendations for future research.

## 2 Background and related work

### 2.1 Event-based signal encoding

The objective of event-based encoding is to create a framework for efficient representation of a continuous-time signal by capturing its values only at important instants (events), rather than registering it at regular time intervals as in classical methods of signal discretization. The event rate depends on the nature of the input signal, growing for example when the input changes substantially, and becoming smaller during periods of low activity. This allows for minimizing the number of events required to accurately represent the signal, reducing unnecessary computations and data transmissions (Miśkowicz, 2003).

The efficient event-based signal representation preserves signal characteristics by retaining its original key features, such as dynamics and frequency content, while avoiding artifacts or noise contribution that can cause generation of the events too frequently. With a flexible definition of events, event-based encoding of continuous-time signals can meet diverse system design objectives and application needs. Most definitions refer to the event as to a change of the state of an object occurring at an instant (Miśkowicz, 2015).

**Figure 1:**
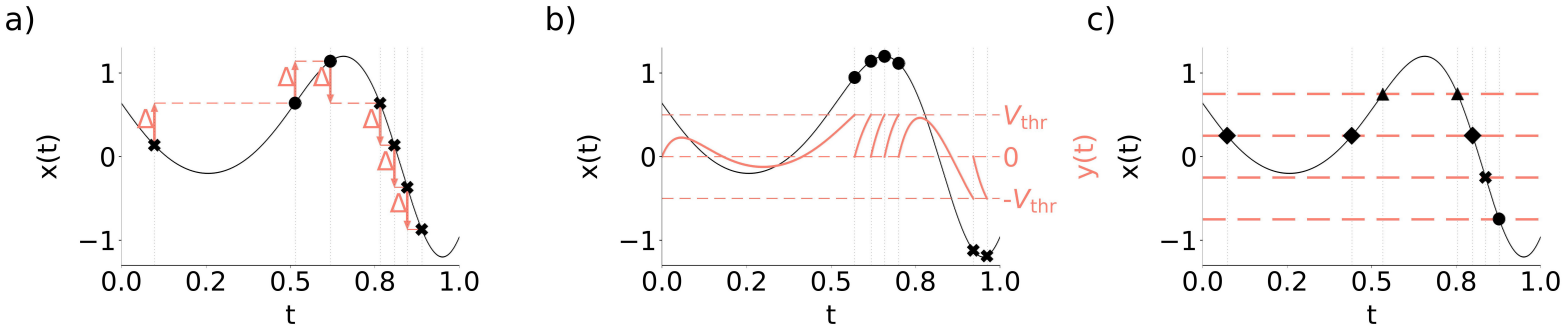
Examples of event-based encoding criteria. Black marker symbols denote different event types produced by the encoding. **(a)** Send-on-delta. **(b)** Leaky integrate-and-fire. **(c)** Level-crossing encoding.

A class of event-based encoding that may be referred literally to the concept of the event as a significant change of the particular signal parameter are *threshold-based criteria*, known in the context of communication as the *send-on-delta* encoding (Miśkowicz, 2006). In the *send-on-delta* scheme, an input signal is encoded as an event when it deviates by a certain threshold (Δ) (Figure 1a). If *x*(*t*) is a continuous-time signal to be encoded, the send-on-delta encoding times *t*_*n*_≥0 for *n* = 1, 2, …  with the threshold Δ are determined such that the following condition holds:


(1) $$\begin{array}{l} t_{n}=\min\left\{t>t_{n-1}:|x\left(t\right)-x\left(t_{n-1}\right)|=\Delta \right\}. \end{array}$$

The size of the threshold Δ defines a trade-off between encoding resolution and the event rate. The threshold can be fixed or modified during system operation depending on the safety and/or performance requirements of the system (Tang et al., 2025), or as a response to variation of a signal slope (Trakimas and Sonkusale, 2011; Wang et al., 2018).

A different integration-based encoding scheme is the integrate-and-fire (IF) encoder that mimics the behavior of biological neurons by describing how a potential of a neuron membrane changes in response to input stimuli (Moser et al., 2025). The IF encoder accumulates an input over time until the total input reaches a threshold *V*_thr_, which causes emission of a spike and a reset of the accumulator to zero:


(2) $$\begin{array}{l} t_{n}=\min\left\{t>t_{n-1}:\frac{1}{\tau_{\text{int}}}\int_{t_{n-1}}^{t}x\left(t\right)dt=V_{\text{thr}}\right\}, \end{array}$$

where τ_int_ is the integration constant.

In a more biologically realistic neuron model, called leaky integrate-and-fire (LIF) encoder, the content of the accumulator decays exponentially with the time constant τ_leak_ (Figure 1b) (Thao et al., 2023):


(3) $$\begin{array}{l} t_{n}=\min\left\{t>t_{n-1}:\frac{1}{\tau_{\text{int}}}\int_{t_{n-1}}^{t}\exp\left(-\frac{t}{\tau_{\text{leak}}}\right)x\left(t\right)dt=V_{\text{thr}}\right\}. \end{array}$$

Another generic class of event-based encoding relevant to many applications in event-based signal processing is not related to detection of an explicit change of the input but focused on matching a certain reference. In the *reference-crossing encoding*, the input is registered when it crosses a prespecified function. The examples are the sine-wave crossing (Selva, 2012) or level-crossing encoding (Mark and Todd, 1981).

In the level-crossing encoding, the reference function can be defined as a single level (Abrahams, 1986), or multiple reference levels (*L*_1_, …, *L*_*m*_) disposed in the amplitude domain (Mark and Todd, 1981) (Figure 1c).

The level-crossing encoding with the set of levels *L*_1_, …, *L*_*m*_ is defined as follows:


$$t_{n}=\min\;\{t>t_{n-1}:$$

(4) $$\left\{\begin{array}{l} x(t)=L_{k}\text{or }x(t)=L_{k+1}\text{if }x^{\prime }(t_{n-1})>0\\ x(t)=L_{k}\text{or }x(t)=L_{k-1}\text{if }x^{\prime }(t_{n-1})<0 \end{array}\right\},$$

where *L*_*k*_ and *L*_*k*+1_ are reference levels, *L*_*k*+1_>*L*_*k*_, 1 < *k*<*m*.

This condition specifies that the next level crossing after the actual crossing of *L*_*k*_ will occur for the same level (*L*_*k*_) or the higher level (*L*_*k*+1_) in case of the up-crossing ($x^{\prime }\left(t_{n-1}\right)>0$) of *L*_*k*_ at *t*_*n*−1_, or for the same level (*L*_*k*_) or the lower level (*L*_*k*−1_) in case of the down-crossing ($x^{\prime }\left(t_{n-1}\right)<0$) of *L*_*k*_ at *t*_*n*−1_.

The level-crossing encodings provide not only time instants when the relevant reference levels are crossed but also imply that the signal remains between consecutive levels during the time between the level crossings (Figure 1c). This means that for *t*∈(*t*_*n*−1_, *t*_*n*_):


(5) $$\begin{array}{ll} L_{k}<x\left(t\right)<L_{k+1} & \text{if}x^{\prime }\left(t_{n-1}\right)>0,\\ L_{k-1}<x\left(t\right)<L_{k} & \text{if}x^{\prime }\left(t_{n-1}\right)<0. \end{array}$$

The extra knowledge defined by the above inequalities, called the implicit information, implies that no event occurs between consecutive encoding instants (Rzepka et al., 2018). The implicit information is relevant not only to the level-crossing encodings but also to any event-based encoding scheme.

The reference levels in the level-crossing encoding are usually uniformly distributed in the amplitude domain although adaptive (Senay et al., 2010) or optimal distributions of levels (Kozat et al., 2013) have been also proposed.

The mean rate of level-crossing and send-on-delta encodings depends on the average slope of the signal (Miśkowicz, 2006), while the rate of spikes in IF encodings is defined by the average values of the input signal. If the hysteresis is adopted to triggering (multiple) level crossings (i.e., the repeated crossings of the same level are not encoded), then such level-crossing criterion coincides with the threshold-based encoding (send-on-delta scheme).

The general model of threshold-based encoding that provides a unified framework for send-on-delta, leaky integrate-and-fire, and other threshold-based schemes (e.g., leaky send-on-delta) has been introduced in Moser (2017).

The threshold-based event encoding is a fundamental paradigm in event-based control (Heemels et al., 2012; Miśkowicz, 2015) and state estimation (Ge et al., 2020) designed for efficient utilization of computation and communication resources, especially when implemented with wireless connectivity in IIoT (Aranda-Escolástico et al., 2024; Miśkowicz, 2006). The send-on-delta encoding is applied to event sensors that mimic the function of eyes (silicon retina), ears (silicon cochlea), nose (e-nose), or touch (e-skin) and emit events when a signal representing relevant modality (vision, sound, olfaction, touch) changes by a prespecified threshold (Tayarani-Najaran and Schmuker, 2021; Cheng et al., 2019). On the other hand, the level-crossing encoding is an effective signal representation in energy-efficient signal processing applications (e.g., ECG analysis with wearable devices) (Ravanshad et al., 2013).

Integrate-and-fire encoders as simplified models of neuronal activity are foundational in computational neuroscience and extensively used in the development of SNNs (Thao et al., 2023; Moser et al., 2025). The SNNs are particularly well-suited for processing temporal data due to their inherent ability to handle time-dependent information (e.g., speech recognition, real-time sensory processing, and dynamic vision) (Nunes et al., 2022). One of the active research areas focuses on improving the efficiency of encoding techniques to enhance the performance and applicability of SNNs in various domains (Auge et al., 2021).

A recent work (Zanoli et al., 2025) has shown that optimization-based level-crossing encoding parameter selection methodologies achieve better signal reconstruction in ECG signal monitoring. In this paper, we analyze signal-to-event encoding schemes by examining how signal representation affects the performance of a machine learning model that processes event data using an event distance measure within the Bayesian optimization process. The analysis of event-based encoding schemes is conducted using sensor data for vehicle classification in an intelligent transportation system.

### 2.2 Spiking neural networks (SNN)

Event stream data can be processed by specialized machine learning models such as the spiking neural networks (SNN). These models process data using impulses that propagate asynchronously through the network (Pfeiffer and Pfeil, 2018). This approach allows SNNs to mimic biological networks more closely than traditional neural networks. While the development of the training rules for SNNs is an active area of research, several major training paradigms have been identified: plasticity-aware training (Falez et al., 2019; Mozafari et al., 2019); network conversion, which maps each component of the source network to its spiking equivalent (Rueckauer et al., 2017; Midya et al., 2019; Stöckl and Maass, 2021); and training with backpropagation (Lee et al., 2016; Wu et al., 2018; Rasmussen, 2019). The latter approach leverages existing algorithms and best practices developed for deep learning with nonspiking neural networks.

Backpropagation-based training of SNNs must address the challenge that the spike-generating function is non-differentiable. The methods to overcome this problem are of two types, depending on how much information from the forward pass is needed to compute the surrogate gradient signal. In event-driven learning the error is propagated only through spikes (Zhang and Li, 2020; Wunderlich and Pehle, 2021; Zhu et al., 2022). In contrast, in a learning scheme similar to that used in the Recurrent Neural Networks (RNNs) the error information is propagated also through time steps which did not elicit a spike (Wu et al., 2018; Neftci et al., 2019; Xing et al., 2020; Bauer et al., 2023). Training models with these methods usually requires simulating the state of the entire network over a finite time window with a fixed time step. However, it is possible to design learning rules that do not require such extensive simulation. One example is the time-to-first-spike SNN (Mostafa, 2018; Kheradpisheh and Masquelier, 2020; Zhou et al., 2021). This model is trained using locally exact gradients of the spike-generating function, making both the forward- and backward-pass through the network event-centric. Recently, the time-to-first-spike SNN model has been extended to the scenario where each postsynaptic neuron can generate multiple events in response to observed spike trains (Pabian et al., 2024).

### 2.3 Inductive loop vehicle magnetic profiles (VMP)

The goal of Intelligent Transportation Systems (ITS) is to enhance the efficient use of existing transportation infrastructure by leveraging accurate traffic data acquisition technologies to monitor and manage traffic flow. Despite recent advancements in sensor technology, inductive loop (IL) sensors remain the most widely used in modern traffic control systems (Klein et al., 2006). This technology features low installation costs, a long lifespan due to its simple design, robustness to weather conditions such as rain, fog or snow, and a flexible architecture that can be adapted to various applications. Systems based on IL sensors are capable of vehicle classification (Gajda et al., 2001; Oh et al., 2002; Ki and Baik, 2006; Jeng and Ritchie, 2008; Meta and Cinsdikici, 2010), vehicle re-identification and tracking (Kwon, 2005; Ndoye et al., 2011; Guilbert et al., 2013, 2014), speed estimation (Sun and Ritchie, 1999; Coifman et al., 2003; Lu et al., 2012), as well as wheel and axle detection (Gajda et al., 2012; Marszałek et al., 2011, 2015, 2018).

At minimum, an IL detector consists of two components: a wire loop with one or more turns mounted on or embedded in the roadway pavement, and a controller cabinet that houses an electronics unit (Klein et al., 2006). When a ferromagnetic or conducting metallic mass passes over the IL connected to a conditioning circuit, the currents induced in the object alter the magnetic field distribution affecting the loop inductance and resistance (Mocholí-Salcedo et al., 2017). These dynamic interactions between the loop and the vehicle can be monitored and analyzed as impedance changes, generating a waveform signal known as the vehicle magnetic profile (VMP), where R-VMP represents its resistive component and X-VMP its reactive component.

**Figure 2:**
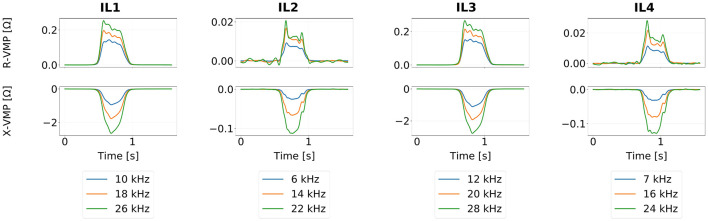
Exemplary VMP profiles acquired by the quad loop VMP measurement system, divided into separate IL sensors, VMP components and loop excitation frequencies. Note that signals originating from the IL sensors of the same type (IL1-IL3; IL2-IL4) are slightly shifted in time. Furthermore, the loop excitation frequency impacts the signal magnitude, but does not significantly change its characteristic features.

Recently, a quad loop, four-channel VMP measurement system was proposed by Marszałek and Duda (2020). It consists of two standard loops (IL1, IL3) and two slim loops (IL2, IL4) arranged in series. The standard loops are well-suited for general-purpose tasks, while the slim loops are preferable for axle identification and wheel rim detection (Gajda et al., 2012; Marszałek and Sroka, 2016). Using two loops of each type instead of a single one introduces redundancy enhancing the system robustness against signal interference and simplifying the estimation of traffic parameters, such as vehicle speed (Mocholí Belenguer et al., 2019). Additionally, three different excitation frequencies are applied to each channel. The simultaneous multi-frequency measurement increases the system resilience to noise, reducing the risk of poor signal quality affecting downstream processing tasks. Figure 2 presents exemplary VMP profiles acquired by this measurement system. We use the quad loop sensor VMP signals originally discretized at 1 kHz in our event-driven signal analysis. A detailed description of the VMP data used in this study is provided in Section 4.

## 3 Materials and methods

In this section we describe the proposed generic framework for encoding parameter selection, which is the main contribution of this paper. Furthermore, in order to show that the parameters selected by the proposed method can be reused when training more complex models, we also introduce the SNN model used in the experimental validation study.

### 3.1 Model-agnostic signal-to-event encoding parameter selection

The main goal of this paper is to identify a set of signal-to-event encoding parameters that preserve information relevant to a multiple event classification task. We operate under the assumption that, for a simple classification model such as the k-NN classifier, the choice of spike encoding parameters has a measurable impact on classification performance and, intuitively, on the pairwise similarity of the generated event data. Consequently, event data that can be accurately classified by the k-NN model are expected to perform well as input to other event-based classifiers, assuming a positive correlation in their outcomes. This makes our method model-agnostic as it does not rely on any assumptions regarding the specific classifier to be used.

**Figure 3:**
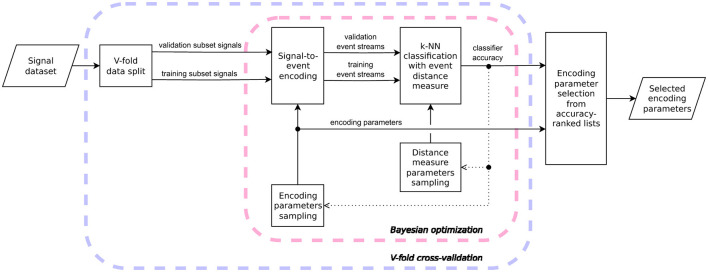
Flowchart of the proposed model-agnostic signal-to-event encoding parameter selection. The k-NN classifier accuracy obtained at various optimization steps is aggregated into ranked lists, along with the corresponding encoding parameters used to encode signals into their event-based representation. The outer loop iterates over different data splits (V-fold cross-validation), while the inner loop performs sampling of encoding parameters for each split using Bayesian optimization. The dotted line representing classifier accuracy indicates that this value serves as the objective function, guiding the parameter sampling in the subsequent iteration of the Bayesian optimization process.

Figure 3 illustrates the proposed methodology for selecting signal-to-event encoding parameters. The approach involves performing V-fold cross-validation using a k-NN classifier that leverages an event-based distance metric to classify signals from the validation subsets. Within each data fold, encoding parameters are sampled via a Bayesian optimization process, guided by the classification accuracy observed in prior iterations. The resulting scores are aggregated into ranked lists (one per data fold) along with their corresponding encoding parameters. The final set of encoding parameters is then selected based on these rankings. Detailed descriptions of the proposed methodology are provided in Sections 3.1.1-3.1.3. It is important to note that if the distance metric used by the k-NN classifier is itself parameterized, its impact on the classification accuracy must be marginalized, e.g., by sampling multiple distance parameter values for each fixed set of signal encoding parameters during the Bayesian optimization process.

#### 3.1.1 Van Rossum distance

A crucial component of the strategy for selection of signal-to-event encoding parameters based on the k-NN classifier is the choice of an appropriate distance metric to assess the similarity between event sequences. The choice of the distance measure heavily depends on the properties of the original signal. For relatively short signals (such as the VMP), typically spanning only a few seconds, the distance measures commonly used in computational neuroscience are well-suited for processing this type of data (Kass et al., 2014).

Due to its prominence in neuroscience, we opt to use the multi-neuron van Rossum distance (van Rossum, 2001; Houghton and Sen, 2008) to assess the similarity of the event sequences produced by spike-encoding functions^1^. It is a purely deterministic distance measure and does not take into account the stochastic nature of event data. Furthermore, it assumes that there is no functional dependence between events from different channels.

Let $U=\left\{{\text{u}}^{1},{\text{u}}^{2},\ldots ,{\text{u}}^{P}\right\}$ and $V=\left\{{\text{v}}^{1},{\text{v}}^{2},\ldots ,{\text{v}}^{P}\right\}$ be two populations of *P* independent neurons. Furthermore, let


(6) $$\begin{array}{l} f\left(t,{\text{u}}^{p}\right)=\underset{m}{\sum }h\left(t-u_{m}^{p}\right),p=1,2,\ldots ,P \end{array}$$

be a spike train with the event sequence $\left\{u_{m}^{p}\right\}$ in the *p*-th spike train of the population U. This spike train is smoothed by a causal exponential kernel


(7) $$h(t;\tau )=\{\begin{array}{ll} 0 & t<0\\ e^{-t/\tau } & t\geq 0 \end{array}.$$

The spike train *f*(*t*, **v**^*p*^) with the event sequence $\left\{v_{n}^{p}\right\}$ is defined analogously. Note that the event sequences $\left\{u_{m}^{p}\right\},\left\{v_{n}^{p}\right\}$ need not have the same number of events. Then, the multi-neuron van Rossum distance (Houghton and Kreuz, 2012) between U and V is


(8) $$\begin{array}{l} d\left(U,V;\tau ,c\right)=\sqrt{\overset{P}{\underset{p=1}{\sum }}\left(R_{p}+c\underset{q\neq p}{\sum }R_{pq}\right)}, \end{array}$$

with


(9) $$\begin{array}{l} R_{p}=\underset{i,j}{\sum }\left(e^{-|u_{i}^{p}-u_{j}^{p}|/\tau }+e^{-|v_{i}^{p}-v_{j}^{p}|/\tau }-2e^{-|u_{i}^{p}-v_{j}^{p}|/\tau }\right) \end{array}$$

being the *labeled line* term representing the single-neuron van Rossum distance (i.e., a distance between neurons that directly correspond to one another), and


(10) $$\begin{array}{l} R_{pq}=\underset{i,j}{\sum }\left(e^{-|u_{i}^{p}-u_{j}^{q}|/\tau }+e^{-|v_{i}^{p}-v_{j}^{q}|/\tau }-e^{-|u_{i}^{p}-v_{j}^{q}|/\tau }-e^{-|v_{i}^{p}-u_{j}^{q}|/\tau }\right) \end{array}$$

is the *summed population* term representing the cross-neuron distance. Assuming that the sequences in populations U, V are sorted, the computational complexity of Equation 8 is $O\left(P^{2}\mu^{2}\right)$, where μ is the longest event sequence across populations U,V; and can be reduced to $O\left(P^{2}\mu \right)$ using the so-called markage trick (Houghton and Kreuz, 2012). Note that Equations 9, 10 can be computed even when one event sequence is empty while the other is not, as well as when both sequences are empty.

The multi-neuron van Rossum distance defined in Equation 8 is parameterized by *c* and τ, which model various phenomena observed in neuroscience. The mixing parameter 0 ≤ *c* ≤ 1 weighs the importance of treating each neuron separately (*c* = 0) vs. viewing the entire population as a single-unit (*c* = 1). The decay constant τ influences the range of interspike dependencies. For τ → ∞ each individual spike contributes to the distance computed at each subsequent spike. Conversely, for τ → 0 spikes impact only their direct neighborhood with τ = 0 causing the measure to count the number of coinciding events in the spike trains. These properties show the versatility of this distance function. Unfortunately, this also means that the choice of *c*, τ impacts the perceived (dis)similarity of spike trains produced by different spike encoding functions.

#### 3.1.2 Encoding parameter selection with Bayesian optimization

To identify the optimal set of parameters for the encoding functions, we employ Bayesian optimization using the Tree-structured Parzen Estimator (TPE) approach, implemented via the Hyperopt software package (Bergstra et al., 2013). Bayesian optimization is a strategy for locating the extrema of an objective function that is expensive to evaluate. It constructs a surrogate model of the objective function based on prior evaluations at sampled points within the parameter space. This model is iteratively refined with each new observation, and subsequent sampling is guided by an acquisition function derived from the surrogate model. The key distinction between Bayesian optimization and a purely random search lies in the sampling strategy: while random search samples the parameter space uniformly, Bayesian optimization leverages prior knowledge by preferentially sampling regions that are more likely to yield improvements, based on past observations.

For a predetermined training and validation data split, the procedure for selecting signal-to-event encoding parameters using Bayesian optimization can be summarized as follows:

1. Apply min-max normalization to scale each signal individually to the range 0–1. This normalization simplifies the process of selecting encoding parameters by making the resulting event stream independent of the original signal's amplitude range.
2. Define the search space for the parameters of the objective function to be optimized. The dimensionality of the optimization search space depends on the selected spike encoding scheme and the choice of the distance measure. In case of the van Rossum distance the search space must include the parameters τ and *c*, which we constrain to the search space log(τ)~**U**(−1, 1) and *c*~**U**(0, 1), where **U**(*a, b*) denotes the uniform random variable on the interval [*a, b*]. It is important to choose the parameter bounds for the encoding functions such that they are able to produce informative event streams—too few events in the sequence might not carry enough information, whereas too many events might encode contributions from artifacts or noise components in the signal that should be ignored.
3. Define the objective function used in the Bayesian optimization process. This function is the classification accuracy of the k-NN algorithm. For each individual record in the validation dataset, the classifier assigns a class label by identifying *k* = 7 most similar signals from the training dataset based on the multi-neuron van Rossum distance and applying majority voting. The number of the nearest neighbors *k* is fixed to avoid introducing an additional parameter into the optimization search space.
4. Run the Bayesian optimization process over 150 iterations. Each iteration involves sampling a new set of parameters (from the search space defined in step 2), extracting event sequences from the input signal, evaluating the objective function, and updating the surrogate model of the objective function conditioned on these parameters. Note that parameter sets which result in empty event sequences are considered invalid and are excluded from subsequent analysis.

Finally, to assess the robustness of the optimization procedure with respect to the data distribution, we apply the stratified 10-fold cross-validation, resulting in a total of 10 × 150 distinct optimization steps for each spike encoding scheme.

Note that we limit the scope of our optimization process to a single objective: maximizing the k-NN classification accuracy. One might consider using a different random search type algorithm to optimize a multi-objective function that also considers the event density to selectively promote solutions that achieve high classification performance while producing fewer events. We leave this topic for further research.

#### 3.1.3 Weighted median parameter selection strategy

The results of the Bayesian optimization process conducted over V different data splits yield several independent ranked lists *Q* = {*q*_1_, *q*_2_, …, *q*_*V*_}. In each ranking *q*, higher position is assigned to parameter sets that achieve better k-NN classifier scores (ties are permitted). Our objective is to determine a single parameter set (per encoding type) from these rankings that can be reliably used by other multiple event classification algorithms operating on the same data. One possible approach would be to select the parameter set with the highest overall performance across all data splits. However, the absolute score depends not only on the selected parameters—which may not have been selected in other splits during the Bayesian optimization^2^—but also on the specific stratified data sample. Moreover, it is preferable to identify the parameter set that performs consistently well across all data splits. Therefore, a selection strategy based solely on top-performing scores in individual rankings is inadvisable.

To address these concerns, we propose the *weighted median selection strategy*—a parameter selection method that accounts for the local (i.e., within-ranking) rank of each result across all data splits. The approach is outlined in Algorithm 1. Each set of parameters sampled during the Bayesian optimization process is assigned a rank reciprocal score *r* based on its position in the corresponding ranking *q*. The ranking lists is then aggregated, and the rank-reciprocal scores are normalized to produce a weight vector **w**_α_, which assigns a weight to each sampled parameter set. This weight vector is used to compute a weighted median of the rank reciprocal scores, defining the final set of encoding parameters returned by the proposed method. If multiple parameter sets share the same rank-reciprocal score equal to the weighted median, the final selection is the median of all such sets. Overall, this strategy fulfills the objective of selecting a parameter set that performs consistently well across different data splits. It achieves this by mitigating the influence of outliers in individual rankings and by emphasizing configurations associated with higher k-NN classifier accuracy through the use of rank-reciprocal weighting.

**Algorithm 1:**
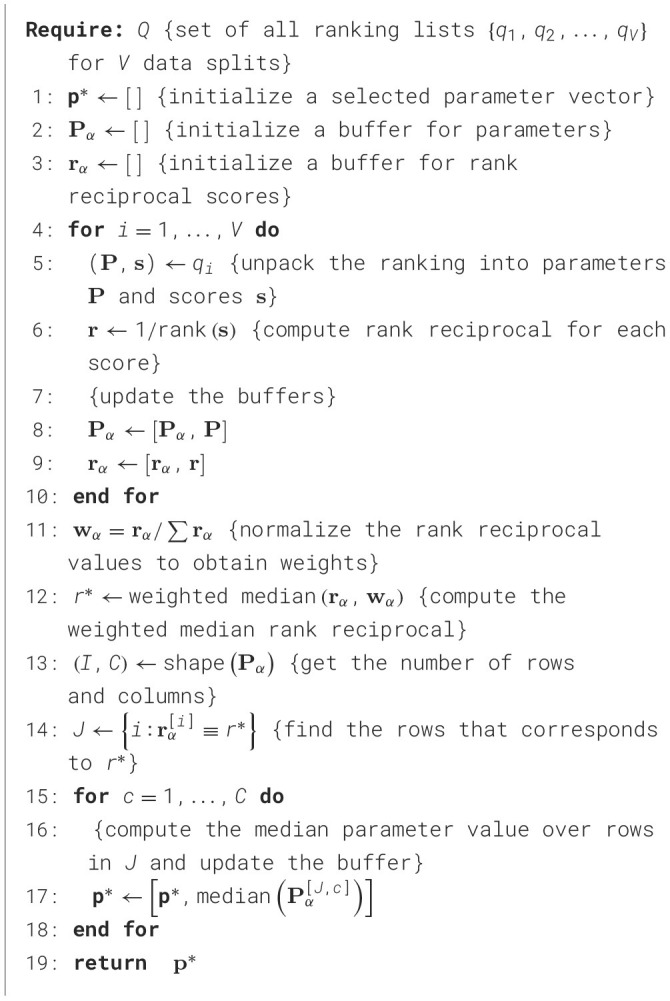
Weighted median selection strategy for the parameter sets found by the Bayesian optimization procedure.

#### 3.1.4 Computational complexity and problem-specific adaptations

It is important to note that the proposed methodology might require problem-specific adjustments as one cannot guarantee that the optimization hyperparameters related to the number of iterations, the number of data splits in the V-fold cross-validation, or the number of nearest neighbors in the k-NN algorithm suits all possible research problems. Fortunately, adjusting these settings does not meaningfully change the proposed methodology. Furthermore, the entire approach is modular enough that it is possible to make bigger changes to some parts of the algorithm without impacting the general framework summarized in Figure 3. We have already mentioned that there might be some benefit to using a different distance measure than the van Rossum distance, one that is better suited to the data under analysis; or to using a different algorithm for the Bayesian optimization procedure.

The computational complexity of the proposed method depends on three main components: the distance measure computation (as established in Section 3.1.2 the van Rossum distance has the complexity $O\left(P^{2}\mu \right)$, where *P* is the number of channels of the signal and μ is the number of events in longest event sequence), the iteration over data points in k-NN classification (with a brute force algorithm complexity of $O\left(N_{t}N_{v}k\right)$, where *N*_*t*_ is the number of training samples and *N*_*v*_ is the number of validation samples in the given data split), and the choice of the signal-to-spike encoding function. This means that for some large-scale problems (e.g., when the dataset has many examples or the signal varies enough that the produced event sequences have numerous events) the proposed methodology might be too expensive to compute and an alternative approach should be devised. Note that the signal must be encoded as an event stream regardless of the chosen optimization method, hence its contribution to the computational complexity analysis should be excluded from comparison.

### 3.2 Time-to-first-spike SNN classifier

To assess how the set of encoding parameters selected by our methodology can be reused to train a more sophisticated event classification model, we train a spiking neural network. Specifically, we use the multiple-input, multiple-output (MIMO) time-to-first-spike SNN introduced by Pabian et al. (2024). This model extends the work by Mostafa (2018) by relaxing the implicit assumption of an infinitely-long refractory period τ_ref_.

We introduce a *neuron response delay factor* τ_delay_ in the first hidden layer of the MIMO SNN. This parameter defines the earliest time at which a given neuron is capable of responding to input events. Functionally, this is equivalent to initializing the neuron in a refractory state lasting τ_delay_. We hypothesize that assigning a different τ_delay_ value to each neuron in the layer enables them to observe slightly different event sequences, thereby mitigating the issue of early events disproportionally influencing the training process. Let us consider the following scenario: assume that all neurons start in a resting state, i.e., τ_delay_ = 0 for all neurons. Additionally, assume that one neuron becomes specialized through training to detect a pattern occurring relatively late in the input event sequence. In this case, this neuron must either adopt smaller synaptic weights to avoid firing prematurely, or maintain comparable weights to other neurons, while producing spikes that are not informative (i.e., spikes triggered by early, irrelevant events). Such behavior arises when a neuron reacts to parts of the input sequence that are not aligned with its intended function.

**Figure 4:**
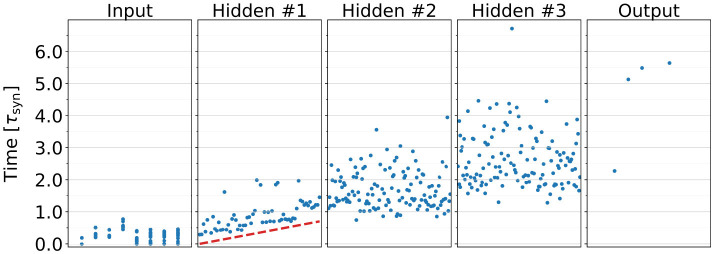
Spike raster plot of a MIMO SNN model trained with a nonzero neuron response delay factor τ_delay_ in the first hidden layer. The dashed line denotes the τ_delay_ value assigned to each neuron in the layer. It is evident that neurons with shorter response delays elicit their first spikes earlier than those with longer τ_delay_ values.

Figure 4 presents an example raster plot of a network trained with nonzero τ_delay_ values assigned to neurons in the first layer, increasing linearly across the layer. We do not apply τ_delay_ to neurons in deeper layers as the SNN-generated event sequences contain significantly fewer events than the network input, simplifying neuron specialization in those layers.

The MIMO SNN models are trained by minimizing the following risk function


(11) $$\begin{array}{l} L_{\text{total}}\left(z,y\right)=\frac{1}{N}\overset{N}{\underset{n=1}{\sum }}L_{n}\left(z,y\right)+\gamma R_{\text{spiking}}+\lambda R_{L_{2}}, \end{array}$$

where


(12) $$\begin{array}{l} L_{n}\left(z,y\right)=-\overset{P}{\underset{p=1}{\sum }}y_{p}^{\left[n\right]}\ln\;\left(\frac{\exp\left(-z_{p}^{\left[n\right]}\right)}{\sum_{p=1}^{P}\exp\left(-z_{p}^{\left[n\right]}\right)}\right), \end{array}$$

is the modified cross-entropy loss for a single example indexed by *n* with:

- *P*- the number of output channels,
- *y*_*p*_ - a binary indicator (0 or 1) of the desired output channel *p* spiking first,
- *z*_*p*_ - the transformed spike time of the *p*-th output channel *z*(*t*) = exp(*t*).

The parameter γ is the synaptic regularization parameter for the spike-firing penalty


(13) $$\begin{array}{l} R_{\text{spiking}}=\overset{H}{\underset{h=1}{\sum }}\overset{M_{h}}{\underset{m=1}{\sum }}R_{mh}, \end{array}$$

where $R_{mh}=\max\left(0,1-\underset{k\in B_{mh}}{\sum }w_{kh}\right)$ with *B*_*mh*_ = {*k*:*t*_*kh*_ < ∞} being the set of valid inputs for the *m*-th output of the *h*-th postsynaptic neuron. Lastly, the parameter λ controls the strength of the *L*_2_ regularization term of the network weights


(14) $$\begin{array}{l} R_{L_{2}}=\underset{i,j}{\sum }w_{ij}^{2}. \end{array}$$

For completeness *R*_*mh*_ = 0 if {*k*:*t*_*kh*_ < ∞} = ∅ . Models are trained with the RMSprop optimizer (Tieleman and Hinton, 2012) with a learning rate of 0.001 over 2,500 iterations with a batch size of 50 examples. We set λ = 10^−5^ in all experiments. Similarly to Pabian et al. (2024), the regularization parameter γ is initially set to a large value of 10^5^ in order to guide the model toward a solution that is capable of propagating event throughout the entire network. Then, after η iterations, the value of γ is decreased to 10^3^ in order to increase the relative importance of solving the classification task.

We use Hyperopt (Bergstra et al., 2013) to optimize the hyperparameters that control how the information is processed by the network. Specifically, the following Bayesian optimization search space was defined:

- the refractory period: log(τ_ref_)~**U**(−0.6, 0),
- the longest neuron response delay τ_delay_ for a linearly-spaced grid: τ_max delay_~**N**(*t*_avg_, 1) subject to τ_max delay_≥0, where **N**(μ, σ^2^) denotes a Gaussian random variable with mean μ and variance σ^2^, and *t*_avg_ is the average event time of input sequences in the current training dataset,
- the number of iterations to train with a larger spike-firing penalty: η~**U**{300, 800}.

This parameter selection process is executed over 30 optimization iterations on a single data split from the V-fold cross-validation. Subsequently, the five best-performing parameter sets are selected and used to train MIMO SNN models on the remaining data splits. In case of tied performance scores, the top-5 Hyperopt models are chosen according to the following heuristic rule: prioritize lower τ_max delay_, higher τ_ref_, and lower η, in that order of importance. This selection strategy favors models that generate fewer events and exhibit faster response time.

## 4 Results

### 4.1 Experimental setup

For this study, we used the same VMP dataset as Marszałek et al. (2023). The dataset consists of 3,328 records categorized into six classes as follows: motorcycle (24.32%), bicycle (20.42%), electric scooter (19.52%), car (15.02%), delivery van (14.11%), and truck (6.61%). Each record contains simultaneous multi-frequency measurements of the real and imaginary components of the VMP signal as described in Section 2.3. For our analysis, we select a subset of channels, retaining only four of them for further processing (the R-VMP signals recorded at the lowest excitation frequency). This choice streamlines our analysis while also encouraging future research that explores the full scope of the available data.

In our analysis of the vehicle classification dataset we consider three encoding types introduced in Section 2.1: level-crossing, send-on-delta and leaky integrate-and-fire (LIF). For the proposed encoding parameter selection with Bayesian optimization we constrain the search space to the following set of probability distributions for each encoding type:

- number of uniformly-distributed amplitude levels of the level-crossing encoding: *L*~**U**{4, 5, …, 16},
- send-on-delta encoding threshold: Δ~**U**(0.02, 0.2),
- LIF encoding parameters: τ_int_~**U**(0.05, 0.4); τ_leak_~**U**(0.2, 0.6); *V*_thr_~**U**(0.2, 0.4).

These constraints were chosen based on preliminary experiments, which showed that certain parameter values resulted in suboptimal event stream densities (e.g., the send-on-delta encoding with Δ>0.2 produced event streams that were too sparse).

Before the signal-to-event encoding, we upsample the digital VMP signals from 1 kHz to 10 kHz in order to more accurately assign the event occurrence time instants. Furthermore, as shown in Figure 2, the individual signals exhibit either all-positive or all-negative amplitudes with only minor variations near the zero baseline. Hence, we take the absolute value of each signal and apply min-max normalization to scale them to the 0–1 range. This normalization eliminates the need to account for variability in amplitude ranges across different VMP sensors. As a side effect of this signal normalization process, the LIF-negative event type cannot occur in the encoded sequences. Additionally, the spike sequences are time-shifted so that the first event within each sequence (across all event types) occurs at a relative time *t* = 0. This adjustment serves two purposes. Firstly, the sensors are positioned in a series, making it essential to preserve the relative time shifts between channels as the vehicle moves over the measurement system. Secondly, any sensor data recorded before the vehicle enters the measurement space should be discarded, enforcing a consistent effective signal start time *t* = 0.

**Figure 5:**
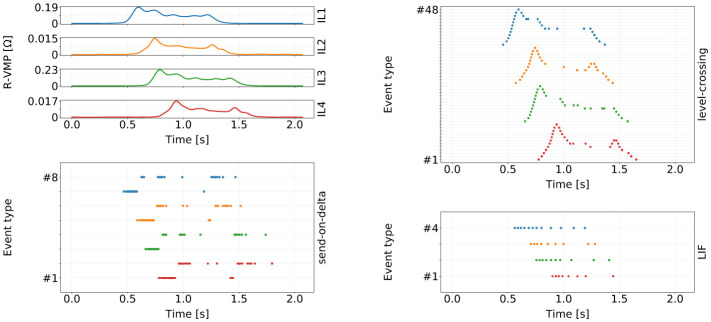
An example of a VMP signal and its different event domain representations. Curves and raster plot points are color-coded to signify the correspondence between the input VMP channel and the output event sequences. The vertical axis for the three encoding raster plots represents different event types: 48 for level-crossing (4 channels × 12 amplitude levels), 8 for send-on-delta, and 4 for the LIF encoding. The number of thresholds in the level-crossing encoding was chosen arbitrarily for illustrative purposes.

Figure 5 shows an example of event sequences obtained for a VMP time series. Each signal-to-event encoding scheme produces a different number of distinct event types for each VMP sensor: level-crossing encoding produces one event type per amplitude threshold; send-on-delta encoding generates two event types (one for “rising” and one for “falling” signal amplitude); LIF encoding produces one event type due to the amplitude normalization step described earlier.

### 4.2 Encoding parameter selection for the VMP dataset

**Figure 6:**
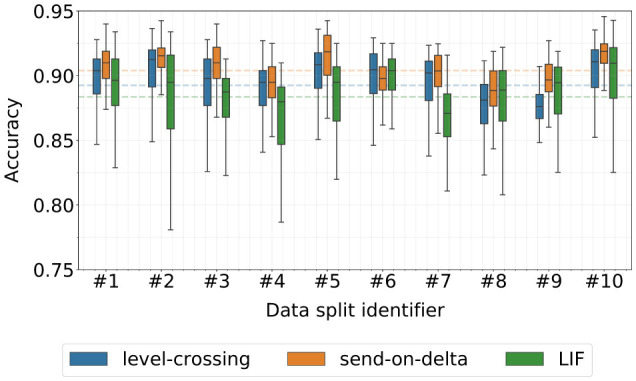
Classification accuracy of k-NN models trained during the stratified 10-fold cross-validated Bayesian optimization procedure. The dashed lines denote the average score over all data splits.

The results of the Hyperopt optimization runs are summarized in Figure 6, and show a wide range of the obtained classifier accuracy spanning approximately from 0.775 to 0.950. This variation highlights the significant impact of encoding parameter selection on the k-NN classifier performance. It is evident that some data splits are more challenging to classify than others, although none of the classifiers deviates significantly from their respective overall-average performance. Furthermore, the results indicate that suboptimal LIF encoding parameters can lead to significantly worse performance than compared to the level-crossing and send-on-delta encoding schemes. In particular, the van Rossum distance parameters were also optimized, influencing the final classification scores. However, determining whether one encoding scheme is definitely superior to the others is not possible based solely on these raw results, due to the factors discussed earlier in Section 3.1.3.

**Figure 7:**
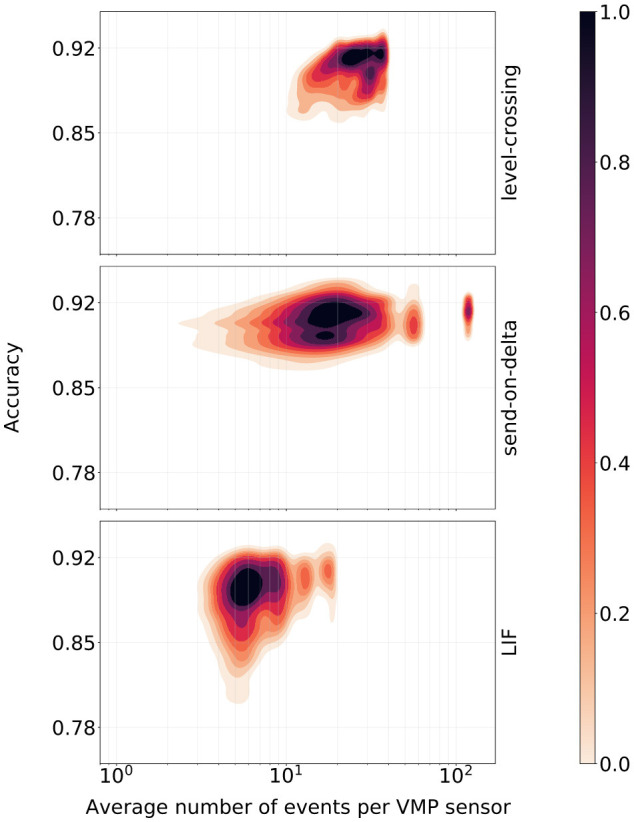
Kernel-smoothed density estimators of the k-NN classifier accuracy scores vs. the average number of events produced by a given encoding scheme per the VMP sensor. Heat maps representing the estimated density were min-max normalized separately.

Additional insights into the encoding schemes were gained by analyzing the classification scores of models trained with Hyperopt in relation to the average number of events produced per VMP sensor^3^. The total number of events in the spike train was normalized by the number of VMP sensors to account for the fact that signals observed by the sensors tend to generate roughly the same number of events after encoding. As a result, the total number of events generated in the system scales linearly with the number of channels, regardless of the selected coding scheme. Additionally, this normalization allows for an assessment of the computational burden associated with adding an additional VMP sensor to the system. The results presented in Figure 7 indicate that the number of events alone is not the sole determinant of classification performance. Specifically, send-on-delta models maintained similar performance regardless of the number of events produced by the encoding scheme. In contrast, different LIF parameter sets, even when generating event sequences of similar length, led to significantly varied k-NN classifier performance.

The selected parameter sets, determined using this strategy, are presented in Table 1, which also includes the stratified 10-fold cross-validated k-NN classifier performance for data splits preprocessed with the chosen encoding schemes. According to these results, level-crossing encoding performs slightly better than the alternatives for the VMP vehicle type classification task. Each signal prior to event encoding contains an average of 1,590 samples. This means that the most data-intensive encoding scheme—send-on-delta—produces a representation that uses approximately 97.8% fewer samples than the original signal.

**Table 1:** Signal-to-event encoding parameters chosen according to the weighted median selection strategy.

**Encoding type**	**Chosen parameters**	**Number of events per VMP sensor**	**Stratified 10-fold cross-validated k-NN performance**	
			**Accuracy**	***F*1-score**
level-crossing	*L* = 12	29.484 ± 8.996	0.912 ± 0.011	0.907 ± 0.016
send-on-delta	Δ = 0.06	36.536 ± 9.040	0.910 ± 0.014	0.909 ± 0.019
LIF	τ_int_ = 0.1	8.317 ± 5.673	0.905 ± 0.011	0.900 ± 0.015
	τ_leak_ = 0.5			
	*V*_thr_ = 0.2			
LIF (*post-hoc* analysis)	τ_int_ = 0.1	20.655 ± 13.768	0.910 ± 0.010	0.906 ± 0.015
	τ_leak_ = 0.35			
	*V*_thr_ = 0.1			

The multi-neuron van Rossum distance parameters used to evaluate the k-NN classifier are omitted. The last row shows the results of a *post-hoc* experiment that repeated the LIF parameter selection analysis with a wider sampling space for the *V*_thr_ parameter and a higher number of the Bayesian optimization iterations. The original result for the LIF encoding parameters was used in subsequent analysis of the SNN classifier training.

**Figure 8:**
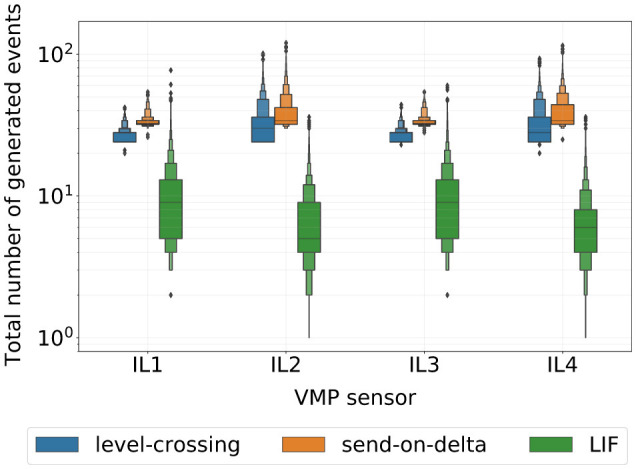
Letter-value plots of the total number of events produced by signal-to-event encoding schemes selected according to the weighted median strategy for each input VMP sensor.

Next, we analyzed the average number of events produced by the selected encoding schemes for each VMP sensor separately. Together with the classification results, this analysis provides insights into the encoding efficiency. Figure 8 summarizes our findings. To visualize these distributions, we use letter-value plots (Hofmann et al., 2017)—an extension of the classical boxplot that more accurately represents distribution tails for large datasets. The results reveal a clear distinction between the empirical distributions of event sequences produced by the two standard inductive loops (IL1 and IL3) and the two slim loops (IL2 and IL4), regardless of the signal-to-event encoding scheme. Additionally, the level-crossing and send-on-delta encoding schemes produce similar overall number of events, with send-on-delta sequences containing slightly more events on average. In contrast, the LIF encoding generates significantly fewer events with the median number of events being approximately three times lower for IL1–IL3 sensors and about six times lower for IL2–IL4 sensors. Moreover, the LIF encoding is capable of adapting to the original signal variability, as evidenced by the broad range of event counts across all data samples. Given that all three classifiers in Table 1 achieve comparable classification accuracy, the LIF encoding appears to be more efficient in terms of the amount of information encoded per event.

Finally, relating the set of selected encoding parameters summarized in Table 1 with the original optimization search space reveals that the proposed method did not trivially select the parameter sets that would maximize the number of events in the event stream sequences. In fact, only the threshold voltage of the LIF encoding was on the sampling space boundary [selected *V*_thr_ = 0.2 for *V*_thr_~**U**(0.2, 0.4)] with all other parameters being selected away from the boundary. This result suggests that the proposed method is not biased in favor of high event density encodings. However, the method itself does not have any safeguards and relies on carefully choosing the parameter bounds to avoid trivial solutions.

Selecting a parameter value for *V*_thr_ that lies on the search space boundary raises a question of whether the parameter space for the LIF encoding has been fully explored. In order to investigate this topic and challenge our initial choice of the search space, we repeated the LIF encoding parameter selection in a *post-hoc* experiment. In this analysis the LIF threshold voltage parameter sampling space was specified as *V*_thr_~**U**(0.05, 0.4). Furthermore, due to an increase in the search space scope, we increased the number of iterations for the Bayesian optimization from 150 to 250. The new set of LIF encoding parameters selected by our method, as well as the average number of generated events and the classifier performance metrics, is presented in Table 1. Not only is the new choice of *V*_thr_ away from the sampling space boundary, but also the k-NN classifier performance for this encoding is on par with the one achieved by the send-on-delta scheme. Additionally, compared to the original result, this new set of LIF encoding parameters more than doubles the number of generated events (on average). Overall, this shows that the proposed method will achieve suboptimal results when the choice of the parameter sampling space for the Bayesian optimization is too constrained. As this result was obtained in a *post-hoc* experiment, we did not use this updated set of parameters of the LIF encoding in subsequent analysis.

### 4.3 Vehicle classification with the MIMO SNN

The set of parameters identified in the previous analysis was used to encode VMP signals into the spiking domain before training the MIMO SNN models for the vehicle type identification task. We applied the same stratified 10-fold data split for cross-validation. The network parameter settings and the hyperparameter optimization grid were described in Section 3.2. In all experiments we used the same *C*-64-128-128-6 architecture, where the number of input neurons *C* depends entirely on the encoding scheme. The specific values of *C* for the encoded VMP data are as follows: *C* = 48 for level-crossing encoding, *C* = 8 for send-on-delta encoding, and *C* = 4 for LIF encoding. This results in a small network with a total of 25, 344+64*C* parameters. However, it is important to note that the effective capacity of the MIMO SNN model is higher than what the raw parameter count suggests, due to the repeated firing of the IF neurons, which are influenced by the refractory period τ_ref_. In total, we optimized the weights of 225 models across the three spike encoding schemes.

**Figure 9:**
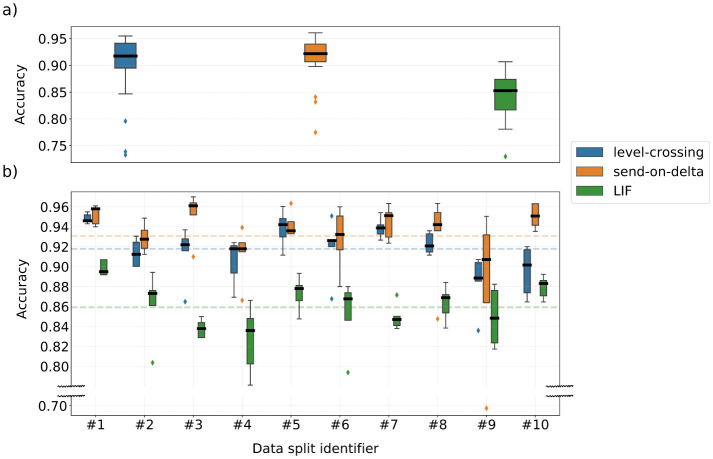
**(a)** Summary of the SNN model performance for models trained with Hyperopt (30 optimization iterations on a single data split of the stratified 10-fold cross-validation). **(b)** Classification accuracy of the SNN models trained with top-5 parameter sets found by the Bayesian optimization process on data split #1. The dashed lines denote the average score over all data splits.

Figure 9a summarizes the classification accuracy of models trained during the Bayesian optimization procedure on a single data split from the stratified 10-fold cross-validation. On average, the LIF encoding performed worse than the alternatives. Recall that all three encoding schemes when parameterized using the weighted median selection strategy achieved similar k-NN classifier accuracy of approximately 0.908 (Table 1). This indicates that the Bayesian optimization procedure failed to find a LIF model that outperformed its own baseline. In contrast, the level-crossing and send-on-delta models performed better than their respective k-NN classifiers, with both achieving similar median accuracy. However, the presence of outliers in the lower accuracy range suggests that poor hyperparameter selection during training can significantly degrade the final model performance. Nonetheless, these results confirm that running the optimization procedure over multiple iterations can lead to a high-performing model, provided that the signal-to-event encoding is chosen correctly.

Figure 9b presents the classification accuracy for models trained with the top-5 best-performing parameter sets selected by Hyperopt, separately for each encoding type and each cross-validation data split. This analysis evaluated the robustness of classifiers trained with these hyperparameters when applied to different input data. Several differences emerge between models operating on differently encoded data. Once again, LIF models exhibited significantly lower absolute performance compared to the other encoding schemes. Additionally, both level-crossing and LIF models evaluated on data splits #2–10 demonstrated worse classification accuracy than the reference split #1, which was used to determine the top-5 hyperparameter sets. In contrast, send-on-delta model evaluated on data splits #3, #7, #8, and #10 achieved performance levels comparable to scenario #1. This suggests that the hyperparameters selected for send-on-delta encoding are more robust across different training datasets. However, this encoding scheme also exhibited an extreme performance outlier in data split #9, deviating from all other trained models. Overall, these findings indicate that send-on-delta encoding is less sensitive to variations in training data, whereas level-crossing and the LIF encoding require more precise tuning to achieve optimal performance.

Lastly, Table 2 presents the top-5 parameter sets for the three encoding types along with the stratified 10-fold cross-validated performance metrics for models trained with these hyperparameters. Notably, the top-5 parameter sets selected for the send-of-delta and LIF models are significantly more similar to each other than those chosen for the level-crossing encoding. This may suggest that, for this specific task, 30 steps of the Bayesian optimization process are sufficient to converge to a good-enough (local minimum) solution for these two model types, but not enough for the level-crossing scheme. Interestingly, the selected values of τ_max delay_ were smaller for the send-on-delta and LIF models when compared to level-crossing scheme. This suggests that a nonzero τ_max delay_ is more beneficial when the number of neurons in the input layer is large (as in level-crossing encoder) rather than when they generate a large number of events (as in send-on-delta encoder). Finally, recall that the prior on the number of training iterations with a larger spike-firing penalty η was U{300,800}. Almost all of the selected values of η ended up at the higher end of this range. This indicates that training the model for longer with a relatively smaller weight assigned to the task-specific loss leads to better final model performance.

**Table 2:** Summary of the top-5 parameter sets found by the Bayesian optimization process used to train the SNN models for the respective spike encoding type.

**Encoding type**	**Number of events per VMP sensor**	**Top-5 parameter sets**			**Stratified 10-fold cross-validated SNN performance**	
		τ_max delay_	τ_ref_	η	**Accuracy**	***F*1-score**
Level-crossing	29.484 ± 8.996	0.9	1.0000	700	0.926 ± 0.019	0.929 ± 0.018
		1.2	0.2512	400	0.917 ± 0.024	0.920 ± 0.027
		0.9	0.6310	600	0.915 ± 0.035	0.917 ± 0.033
		0.9	0.2512	700	0.919 ± 0.024	0.922 ± 0.024
		0.7	0.7943	500	0.913 ± 0.028	0.919 ± 0.028
Send-on-delta	36.536 ± 9.040	0.3	0.3981	600	0.937 ± 0.030	0.928 ± 0.031
		0.4	0.5012	600	0.926 ± 0.036	0.913 ± 0.044
		0.4	0.5012	700	**0.946 ± 0.018**	**0.937 ± 0.021**
		0.4	0.5012	800	0.937 ± 0.026	0.926 ± 0.027
		0.0	0.3162	500	0.909 ± 0.073	0.907 ± 0.068
LIF	8.317 ± 5.673	0.0	0.5012	300	0.861 ± 0.024	0.859 ± 0.027
		0.2	0.5012	600	0.868 ± 0.031	0.856 ± 0.040
		0.0	0.3981	700	0.854 ± 0.032	0.852 ± 0.038
		0.0	0.5012	400	0.859 ± 0.024	0.858 ± 0.024
		0.0	0.5012	700	0.855 ± 0.031	0.851 ± 0.038

Bold values indicate the best classifier accuracy and F1-score across all experimental settings.

When compared to the performance of the baseline k-NN classifiers (Table 1), training the SNN results in lower error rates for the vehicle type classification task, provided that the signal-to-event encoding scheme is properly selected. While processing an example through the SNN is undoubtedly more computationally demanding than computing the multi-neuron van Rossum distance between a pair of examples, a k-NN-based system prediction time scales poorly with the size of the reference database, making it less efficient as the number of training examples increases.

Considering these factors, the send-on-delta model emerged as the most effective event-based encoding scheme for the VMP-based vehicle classification. This model achieved the highest stratified 10-fold cross-validation performance, demonstrated greater robustness across different data splits, and allowed the Bayesian optimization procedure to converge to a locally optimal solution within the given number of optimization steps. In particular, this cannot be solely attributed to the large average number of events generated by the encoding, as it is comparable to that for the level-crossing event sequences (as shown in Figure 8).

## 5 Discussions and conclusions

We proposed a novel methodology for selecting signal-to-event encoding parameters that preserves critical signal information for multiple event classification tasks. This approach is model-agnostic and independent of the complexity of the classification model that will eventually process the data. By decoupling encoding parameter selection from the hyperparameter tuning phase of model development one can simplify the overall prototyping process. Additionally, this method enables a clearer assessment of how different input encoding parameters influence final model performance, ensuring a more consistent and meaningful comparison across models.

This study evaluated three event-based signal encoding schemes in a vehicle classification task: level-crossing, send-on-delta and leaky integrate-and-fire (LIF) models. The aim of the analysis was to assess the impact of encoding schemes on machine learning performance. Our findings reveal that the accuracy of the k-NN classifier varies significantly, ranging from approximately 0.775 to 0.950. This wide performance gap underscores the critical importance of selecting appropriate encoding parameters. To address this issue, we introduced a *weighted median selection strategy*, which constructs performance ranking lists for each data split and determines the median performance across different hyperparameter settings. This approach enabled us to establish a single optimal parameter set for each encoding scheme, yielding average classification accuracies ranging from 0.905 (for the LIF encoding) to 0.912 (for send-on-delta). These results provide a robust baseline for SNN models, given the comparable cross-validated performance of the three k-NN classifiers. Moreover, the selected encoding parameter sets significantly reduce data redundancy compared to the original digitized signal. The most data-intensive encoding—send-on-delta—still produces approximately 97.8% fewer samples than the original signal representation, demonstrating the efficiency of event-based encoding. All three encoding schemes achieved comparable levels of classification accuracy while producing encodings of different event density. This highlights the importance of testing multiple alternative encoding schemes in order to figure out the one that best satisfies the system design constraints.

For the SNN trained on data encoded by the event-based schemes, the send-on-delta models emerged as the best-performing group of networks. They achieved not only the highest overall cross-validated accuracy (0.946 on average), but also demonstrated greater robustness across different data splits. Additionally, the Bayesian optimization procedure successfully converged to a locally optimal solution within the limited number of optimization steps. However, this was achieved while generating the highest average number of events among the tested signal-to-event encoding schemes, slightly more events than the second-best approach, level-crossing encoding. In contrast, the SNNs trained on time-series data encoded using the LIF scheme performed significantly worse than the tested alternatives. We hypothesize that three key factors contributed to this outcome: the small number of distinct event types produced by the encoding (only one per input sensor), the relatively low number of events observed by the network (approximately three to six times fewer than in the alternative schemes), and the complexity of optimizing multiple encoding parameters (compared to a single parameter for send-on-delta and level-crossing schemes). Further investigation is needed to determine whether these factors universally impair the SNN classifier performance, regardless of the encoding type. Note that in a *post-hoc* experiment for the LIF parameter selection a slightly different set of parameters was selected, one that more than doubles the number of generated events (on average). While using this parameter set to train an SNN could have addressed the problem of low event stream density, it would not have helped with the small number of distinct event types produced by the LIF encoding. Nevertheless, the SNN training experiment should be considered as supplementary to the main goal of the paper (choosing an event-based signal encoding parameters) and is not intended to be a commentary on which encoding scheme—if any—is the best. Overall, our findings suggest the presence of a trade-off between classification performance and energy efficiency, measured by the number of generated events. These considerations must be carefully balanced when designing an end-to-end event-centric machine learning solution.

In this paper, we employed a simple multichannel spike train model as defined in Equation 6. This model assumes that there is no functional dependence between events from different channels. However, in certain scenarios, this assumption might not hold, as events from multiple channels can interact. To account for such dependencies, an alternative spike train model can be defined as follows:


$$\begin{array}{ccc} f_{p}\left(t,U\right) & = & \underset{m}{\sum }h\left(t-u_{m}^{p}\right)+\alpha \overset{P}{\underset{\begin{array}{c} s=1\\ s\neq p \end{array}}{\sum }}\underset{k}{\sum }h\left(t-u_{k}^{s}\right), \end{array}$$

(15) $$\begin{array}{c} p=1,2,\ldots ,P, \end{array}$$

where the parameter α>0 controls the strength of the multichannel interactions. If α = 0, the model reduces to the one defined in Equation 6. Extending our learning strategy to this dependent multichannel spike train model presents an interesting option for further research. Additionally, this study could be extended to incorporate other event-based encoding schemes and to train more complex classifiers using the proposed methodology. Finally, our optimization objective considered only the classification accuracy and we made *post hoc* insights into the encoding event density efficiency for parameter sets selected according to such objective. It would be interesting to see a multi-objective optimization approach that also considers this event-based efficiency to allow better control over the trade-off between classification performance and the number of generated samples.

## Data Availability

The data analyzed in this study is subject to the following licenses/restrictions. Data provided by a third-party—the authors of this article do not hold the right to share the data. Requests to access these datasets should be directed to Zbigniew Marszałek, antic@agh.edu.pl.

## Footnotes

^1^The van Rossum distance was originally defined for neural spike responses, therefore its formulation refers to various (populations of) neurons. We adopt this terminology for consistency with previous works in the field, noting that different “neurons” relate to “event types”, whereas “populations of neurons” intuitively correspond to “sets of event types.”

^2^In Bayesian optimization, the sampling process depends on the sequence of scores obtained in previous iterations. Since each data split contains a different subset of examples, it is not possible to guarantee that the same parameter sets will be sampled across splits even if the sampling is pseudo-random, i.e., initialized with a fixed random seed, unless the sequence of scores in successive iterations is identical.

^3^Recall that the term “VMP sensor” can refer to either the physical IL sensor or the excitation frequency of the loop. Throughout our analyses we used four “VMP sensors” representing the R-VMP signals registered by the quad-loop system at a single excitation frequency per loop.
